# Using a Leroux-prior-based conditional autoregression-based strategy to map the short-term association between temperature and bacillary dysentery and its attributable burden in China

**DOI:** 10.3389/fpubh.2024.1297635

**Published:** 2024-05-17

**Authors:** Jianping Wang, Kai Lu, Yuxin Wei, Wei Wang, Yongming Zhou, Jing Zeng, Ying Deng, Tao Zhang, Fei Yin, Yue Ma, Tiejun Shui

**Affiliations:** ^1^West China School of Public Health and West China Fourth Hospital, Sichuan University, Chengdu, China; ^2^Yunnan Center for Disease Control and Prevention, Kunming, China; ^3^Sichuan Center for Disease Control and Prevention, Chengdu, China

**Keywords:** temperature-BD association, meta-analysis, LCAR-based three-stage strategy, bacillary dysentery, spatial heterogeneity, spatial autocorrelation

## Abstract

**Background:**

In China, bacillary dysentery (BD) is the third most frequently reported infectious disease, with the greatest annual incidence rate of 38.03 cases per 10,000 person-years. It is well acknowledged that temperature is associated with BD and the previous studies of temperature-BD association in different provinces of China present a considerable heterogeneity, which may lead to an inaccurate estimation for a region-specific association and incorrect attributable burdens. Meanwhile, the common methods for multi-city studies, such as stratified strategy and meta-analysis, have their own limitations in handling the heterogeneity. Therefore, it is necessary to adopt an appropriate method considering the spatial autocorrelation to accurately characterize the spatial distribution of temperature-BD association and obtain its attributable burden in 31 provinces of China.

**Methods:**

A novel three-stage strategy was adopted. In the first stage, we used the generalized additive model (GAM) model to independently estimate the province-specific association between monthly average temperature (MAT) and BD. In the second stage, the Leroux-prior-based conditional autoregression (LCAR) was used to spatially smooth the association and characterize its spatial distribution. In the third stage, we calculate the attribute BD cases based on a more accurate estimation of association.

**Results:**

The smoothed association curves generally show a higher relative risk with a higher MAT, but some of them have an inverted “V” shape. Meanwhile, the spatial distribution of association indicates that western provinces have a higher relative risk of MAT than eastern provinces with 0.695 and 0.645 on average, respectively. The maximum and minimum total attributable number of cases are 224,257 in Beijing and 88,906 in Hainan, respectively. The average values of each province in the eastern, western, and central areas are approximately 40,991, 42,025, and 26,947, respectively.

**Conclusion:**

Based on the LCAR-based three-stage strategy, we can obtain a more accurate spatial distribution of temperature-BD association and attributable BD cases. Furthermore, the results can help relevant institutions to prevent and control the epidemic of BD efficiently.

## 1 Introduction

Bacillary dysentery (BD), an intestinal infectious disease caused by *Shigella* bacteria, is transmitted by the fecal-oral route through polluted food and water, being in contact with carriers, or inadequate sanitation and hygiene (1). With approximately 188 million cases and 164,300 *Shigella-related* deaths (12.5% of all diarrheal deaths) reported per year worldwide (2), BD has brought considerable concern globally. In China, BD is the third most frequently reported infectious disease, with the greatest annual incidence rate of 38.03 cases per 10,000 person-years (3).

It is well acknowledged that temperature is associated with BD, and investigating the association between temperature and BD can help deepen its epidemiological understanding and formulate temperature-related public health interventions. Numerous time-series studies have investigated the short-term association between temperature and BD, which shows considerable heterogeneity among the provinces in China. For example, a study in Jilin, China (4), showed a “J” curve of the association and positive correlation between temperature and BD. A study in the southern and northern parts of China (5) showed that each 1°C rise in temperature caused a 1.01 and 4.26% increase in BD risk, respectively. This heterogeneity may stem from various analysis methods, yet the more probable explanation is its intrinsic existence among provinces. Furthermore, the results of related studies show that adjacent provinces have similar associations, which indicates the spatial autocorrelation of association instead of a random distribution of heterogeneity.

Ignoring the heterogeneity of associations may lead to inaccurate estimation of temperature-BD association in local regions. This could lead to incorrect assessments of attributable risk and potentially misinform the design of temperature-related public health interventions for preventing and controlling BD. Currently, many studies, such as Zhu’s et al. (6), adopt a common meta-analysis-based two-stage analytical process considering the heterogeneity of association as a random error and ignore the spatial autocorrelation of association among regions, which may lead to an inaccurate result, especially in characterizing the exact spatial distribution of exposure-response relationship of each spatial unit (7). In addition, some studies using the stratified strategy, like Liu’s et al. (8), divide the whole region into several subregions and then make an independent analysis among subregions. However, the division is somewhat subjective, and the number of divided subregions is small to maintain the stability of the estimation. To the best of our knowledge, no study has appropriate consideration of the spatial autocorrelation of association and the attributable burdens when focused on the spatial distribution of the short-term association between temperature and BD.

The latest proposed LCAR-based two-stage strategy (9) provided a reasonable method for mapping the spatially autocorrelated association. In contrast to the meta-analysis-based two-stage strategy, the LCAR-based strategy used the Leroux-prior-based conditional autoregression (LCAR) to replace the meta-regression in the second stage. This approach aims to sufficiently utilize the spatial dependence information. Many studies have demonstrated its capacity to accurately obtain short-term associations (10). In this study, based on the idea, an LCAR-based three-stage strategy was carried out to map the temperature-BD association, and then, the attributable BD burdens were calculated.

Specifically, we chose 31 provinces in China to characterize the potentially heterogeneous association between monthly average temperature (MAT) and BD in the first stage, followed by the LCAR to smooth the spatial distribution of the association in the second stage. Based on the region-specific association, a more accurate BD burden attributed to high temperature was calculated in the third stage. These results can help relevant institutions recognize high-risk areas and reasonably distribute resources for public health and thus effectively prevent and control the incidence of BD.

## 2 Methods

### 2.1 Data

**Figure 1:**
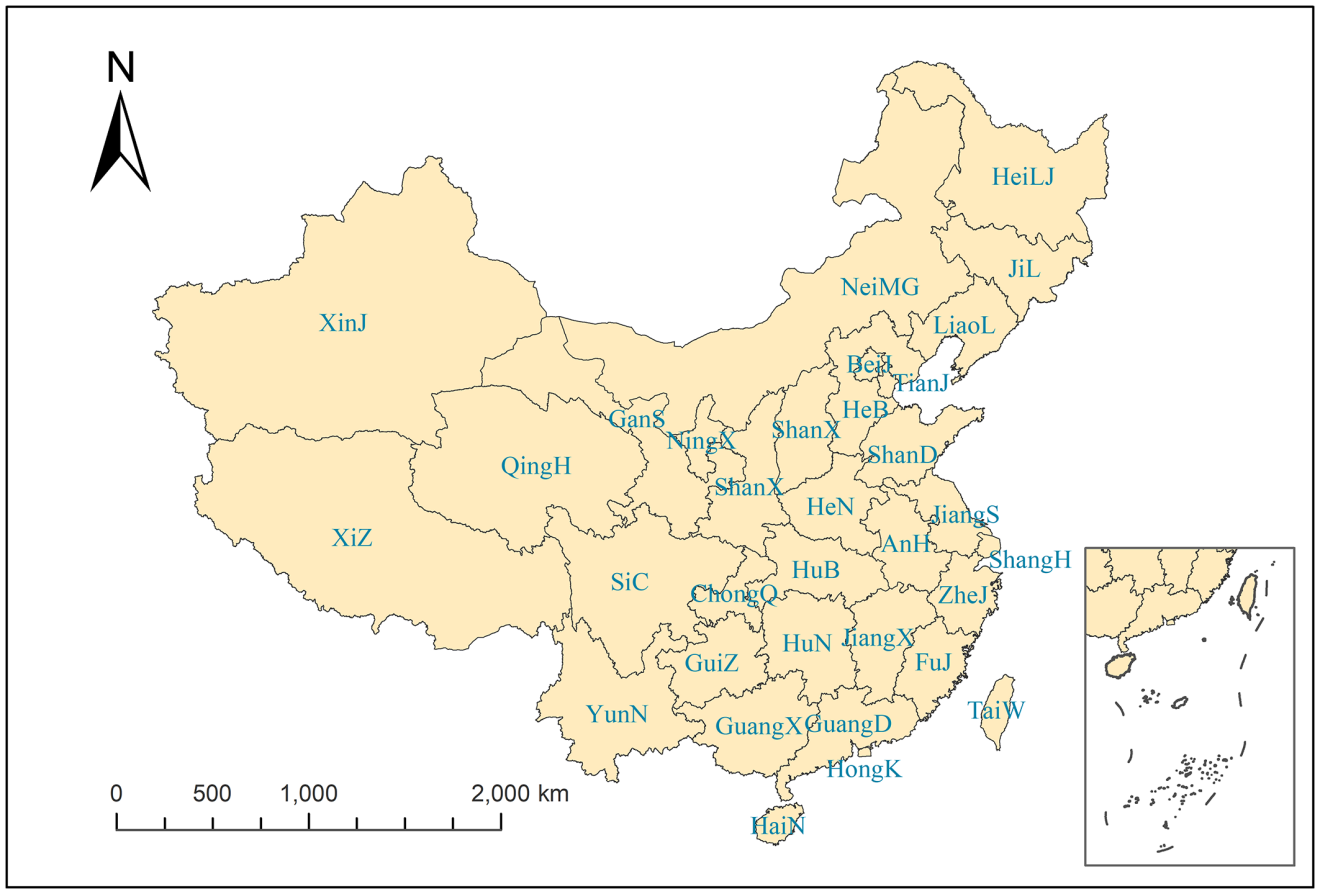
Spatial distribution of study regions.

We chose 31 provinces of China as our study regions, and to exhibit convenience, we adopted the abbreviated names, as shown in the Supplementary material. Figure 1 shows the spatial distribution of our study regions. The monthly province-level numbers of BD cases from 2004 to 2017 were collected, with 3,710,962 cases in total and an average of 71,255 cases per month from the Chinese Center for Disease Control and Prevention.^1^ For the significance of public health, it is more worthwhile to prevent and control infectious diseases on a monthly scale. Meanwhile, the monthly data can mitigate the variation in the number of days per month (11).

The monthly meteorological data were collected from the China Meteorological Data Sharing Service System,^2^ comprising average temperature (°C), mean relative humidity (%), average wind speed (m/s), sunshine duration (h), and average rainfall (mm). We utilized kriging interpolation within ArcGIS 10.2 (ESRI, ArcGIS 10.2, Redlands, CA, United States) to derive daily meteorological data for cities from cleaned weather station data. Subsequently, we computed monthly averages from these daily values. Finally, we applied a population-weighted average to calculate the provincial monthly meteorological data. The population-weighted formula for each province is defined as follows:


$$\bar{x}=\frac{\sum w_{i}x_{i}}{\sum w_{i}},$$

where $\bar{x}$ represents the weighted average of each meteorological factor for the province. $x_{i}$ is the monthly average of each meteorological factor in the city i of the province, and $w_{i}$ is the population of the city i .

### 2.2 Statistical analysis

#### 2.2.1 The first stage: estimating the association between temperature and BD in 31 provinces

For each province, a generalized additive model (GAM) with an identical form was used to independently estimate the province-specific association. The quasi-Poisson likelihood function was chosen to accommodate overdispersion. A natural spline function was utilized to depict the relationship between MAT and BD. Due to the maximum incubation period of BD being shorter than 1 month, the lag effect was not considered. Other meteorological factors, e.g., humidity, rainfall, wind speed, and sunshine duration, were included as confounders. The model was constructed as follows:


$$Y_{it}\sim \mathrm{Quasi}-\mathrm{Poisson}\left(\mu_{it}\right)$$

$$\log\left(\mu_{it}\right)=\mathrm{ns}\left(\mathrm{tm,df}=4\right)+\mathrm{humid}+\mathrm{rain}+\mathrm{wind}+\mathrm{sun}+s\left(\mathrm{time}\right)$$

where $Y_{it}$ denotes the monthly number of BD cases in month t and province i , with $E\left(Y_{it}\right)\equiv \mu_{it}$ . MAT was applied with natural splines (df = 4) for the exposure dimension. The confounders humidity ( humid ), rainfall ( rain ), wind speed ( wind ), and sunshine duration ( sun ) were applied in linear form. s (.) represents the thin plate spline functions, which were applied to adjust for seasonality and long-term trends. The R square and score of generalized cross-validation (GCV) were used to determine the optimal model form, i.e., select appropriate degrees of freedom for ns(.) and s(.). The degree of freedom of the natural splines function was selected by varying from 1 to 8, and we obtained df = 4 based on the R square and the GCV score.

Based on this model, we calculated the logarithm of relative risks (log-RRs) of two types of different temperatures. One type is the absolute MAT 15°C, which is approximately the 50% quantile MAT across all 31 provinces, assuming that the reference temperatures in all provinces are identical. The other type is the relative MAT, where we selected the 50% quantile MAT within each province as its respective reference temperature. For the former, the identical absolute reference MAT remains the intuitive epidemiological explanation and is conducive to assessing BD burden attributable to extreme hot temperatures on a nationwide scale, but for a local region, such as in Xizang, it may be unreasonable to choose 15°C as a reference because the maximum temperature is 16°C, which may lead to the relative risk referring to 15°C a epidemiologically meaningless value in controlling BD and assessing the burden attributable to the region-specific extreme cold or hot temperature. In contrast, relative MAT assumes that the association is consistent in the same percentage of temperature of each province, which can provide a more appropriate reference for a specific province. Ultimately, we obtained the log-RRs with an interval of 0.1°C for absolute MAT and 1% for quantile MAT and calculated its estimate error.

#### 2.2.2 The second stage: estimating spatially smoothed province-specified associations and characterizing their spatial distribution

Based on the log-RRs and its estimated error from the first stage, we adopted LCAR to spatially smooth it to obtain more accurate and stable results in the second stage, which can adequately consider the spatial autocorrelation with a spatial effect term. Meanwhile, the meta-analysis was also adopted to smooth the log-RRs, and we compared the performance of these two models. For each target MAT, the LCAR model is constructed as follows:


$${\hat{\theta }}_{i}=\theta_{i}+\varepsilon_{i}$$

$$\varepsilon_{i}\sim N\left(0,{\hat{\sigma }}_{i}^{2}\right),$$

$$\theta_{i}=\eta +\xi_{i},$$

$$\xi \sim MN\left(0,{\left[\tau W\right]}^{-1}\right),$$

where ${\hat{\theta }}_{i}$ represents the log-RR of the target MAT for province i , which was calculated by the GAM model in the first stage; $\theta_{i}$ is the unknown true value; ${\hat{\sigma }}_{i}^{2}$ is the estimated variance of log-RR in the first stage; and η denotes the average association across all provinces. N(.) and MN(.) represent normal distribution and multivariate normal distribution, respectively. $\xi_{i}$ ’s measure the spatial heterogeneity. τ is a precision parameter. W is an LCAR-based spatial prior matrix that is defined as


$$W=\rho R+\left(1-\rho \right)I_{n\times n},$$

where ρ is a parameter to balance the structured and unstructured spatial random effects. $I_{n\times n}$ is the identity matrix with dimensions of = 31. R is a symmetric matrix that ${\left(R\right)}_{ij}$ = 1 if district i and district j have a common boundary and = 0 otherwise to represent the spatial neighbor relationship, and the diagonal elements are equal to the number of neighbors around the i th province.

In this way, a series of spatially smoothed log-RRs of target MAT were calculated with higher accuracy and stability for 31 provinces.

#### 2.2.3 The third stage: estimating the attributable burden of high temperature on BD and characterizing its spatial distribution

**Figure 2:**
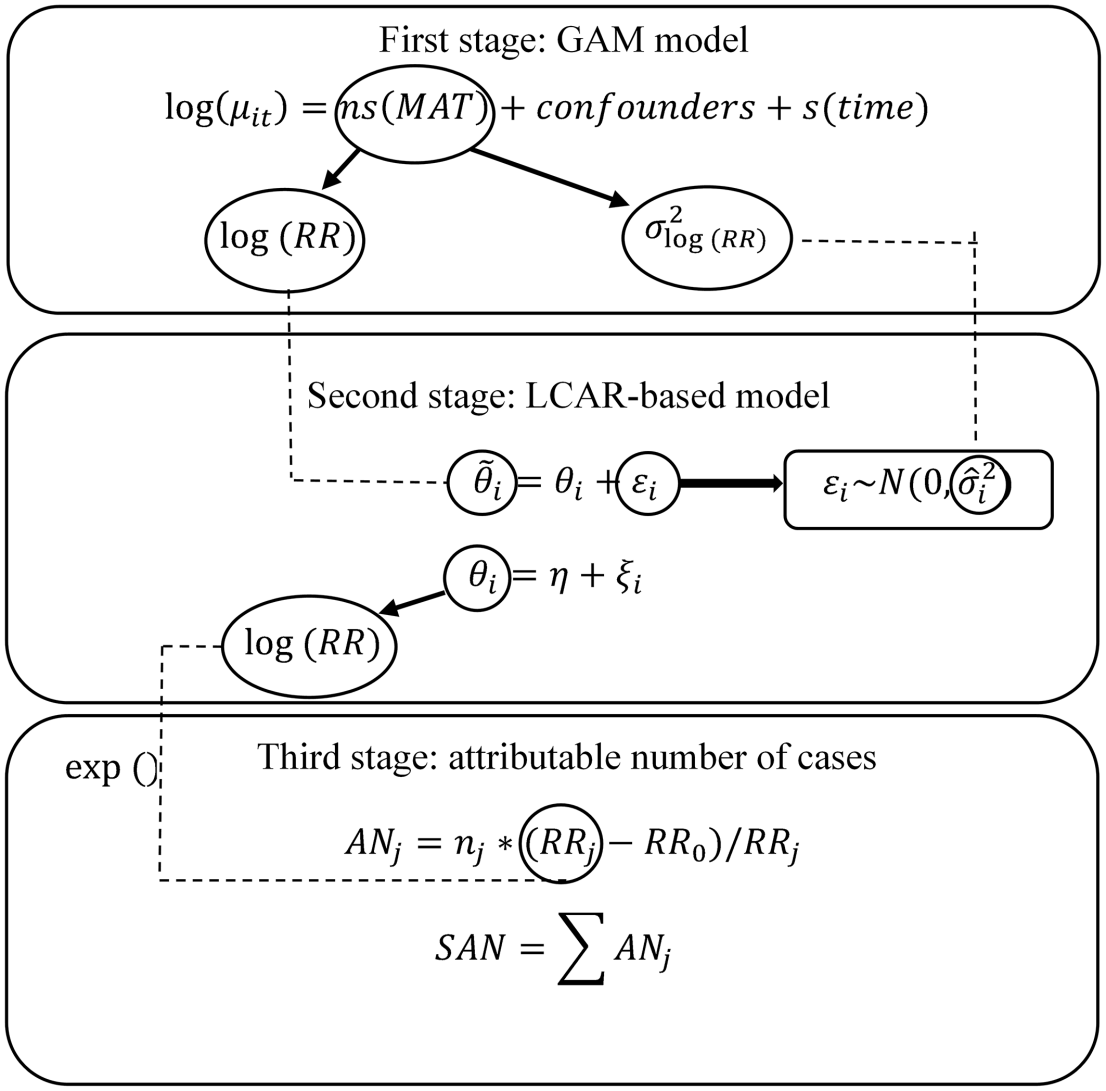
Flowchart of my modeling framework.

Based on the more accurate and stable estimation of relative risk in the second stage, we can calculate the more accurate attributable burden, i.e., attributable BD cases, of high temperature, which was defined as the MAT above the median value. As such, for each province, the attributable burden was calculated as follows:


$${\mathrm{AF}}_{j}=\left({\mathrm{RR}}_{j}-{\mathrm{RR}}_{0}\right)/{\mathrm{RR}}_{j},$$

$${\mathrm{AN}}_{j}=n_{j}*{\mathrm{AF}}_{j},$$

$$\mathrm{SAN}=\sum {\mathrm{AN}}_{j},$$

where j denotes the month with high temperature. ${\mathrm{RR}}_{j}$ denotes the relative risk of MAT in month j , and ${\mathrm{RR}}_{0}$ denotes the relative risk of median MAT in each province, which is equal to 1. $n_{j}$ represents the number of BD cases in month j . Ultimately, we calculate the attribute number of incidence of BD due to high temperature (12–15), i.e., AN , and obtain the total attribute number, i.e., SAN, to reflect the BD disease burden from the temperature for each province and draw a disease map to describe its spatial distribution. Figure 2 shows the whole framework of our analysis.

We adopted a different form of exposure-response and degree of freedom and took the best parameters based on the R square and REML of the model.

All analyses were performed using the “mgcv” and “INLA” packages in R software (version 4.1.2). All statistical tests were two-sided, and we considered *p* < 0.05 to be statistically significant.

## 3 Results

### 3.1 Spatial distribution of meteorological and disease data

**Figure 3:**
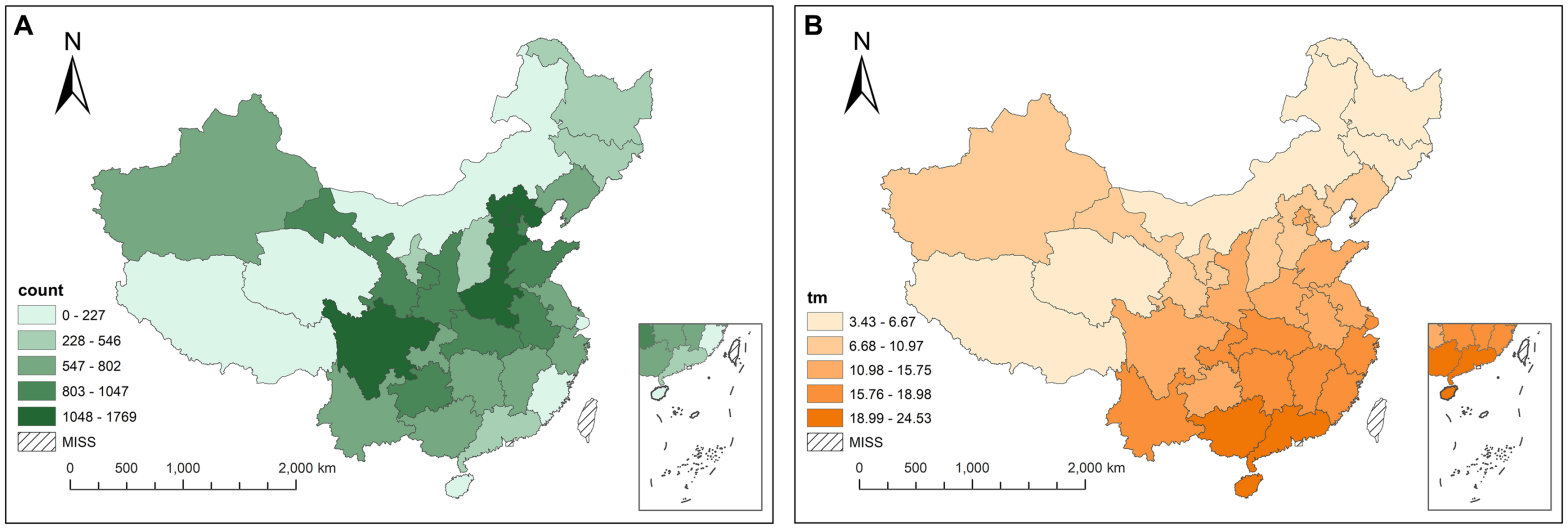
Spatial distribution of the average BD cases **(A)** and average MAT **(B)** in 31 provinces.

**Figure 4:**
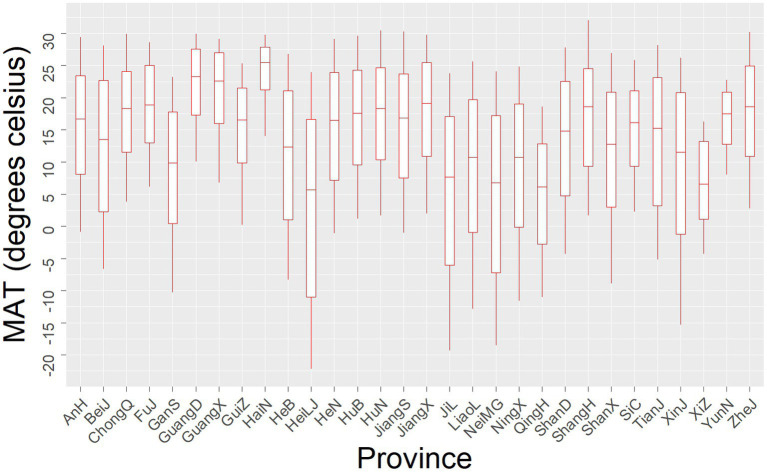
Distribution of MAT in 31 provinces.

A total of 3,710,962 BD cases were reported from January 2004 to December 2017, with a monthly average of 71,255 cases. Figure 3A shows the spatial distribution of the monthly average cases. The largest number of cases recorded in 31 provinces is 297,249 (8%) in total and 1769.33 per month in Beijing, and the smallest is 12,333 (0.3%) in total and 73.41 per month in Hainan. In general, the cases are mainly concentrated in southeastern China, e.g., Sichuan and Henan, which reveals the spatial heterogeneity of the incidence of BD. Figure 3B shows the spatial distribution of the average MAT, which gradually increases from the north to south of China. The range of MAT of 31 provinces is shown in Figure 4. The minimum MAT of Hainan is 14°C, and the maximum MAT of Xizang is 16.3°C.

### 3.2 Spatially smoothed association

**Figure 5:**
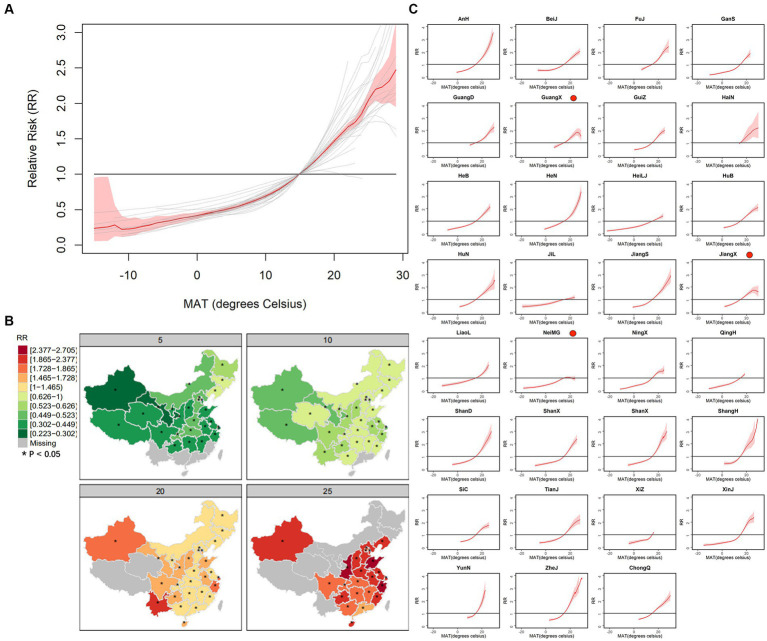
The curves of average association **(A)** and province-specific association between absolute MAT and BD **(C)** and spatial distribution of RRs of some representative absolute MAT, i.e., 5, 10, 20 and 25°C **(B)**.

For the second stage, we adopted LCAR to smooth two types of log-RRs of MAT from minimum to maximum with a separation of 0.1°C. Figure 5 shows the association curves and spatial distribution of RR of absolute MAT. As shown in Figure 5A, the specific exposure-response curves of 31 provinces show the spatial heterogeneity of association: Most curves show a higher relative risk with a higher MAT, but some of them have an inverted “V” shape. Figure 5B shows the spatial distribution of RRs of some representative absolute MATs, i.e., 5, 10, 20, and 25°C, and the colors of western provinces are usually deeper, which reveals that their incidence risks are more sensitive to MAT. The RRs of representative abstract MAT and division of area of 31 provinces can be seen in Supplementary Table S2, and the maximum RR is 2.704 of 20°C in Zhejiang and the minimum RR is 0.432 of 5°C in Xinjiang. The missing areas indicate the provinces whose temperature range does not contain the representative MAT. As shown in Figure 5C, although the curves mainly show a higher relative risk when MAT increases, the curves of Guangxi, Jiangxi, and Inner Mongolia show a lower risk with higher MAT when MAT reaches above 25°C. Furthermore, the curves show a threshold MAT of 10°C, and the growth of curves becomes steeper when the MAT reaches above it.

**Figure 6:**
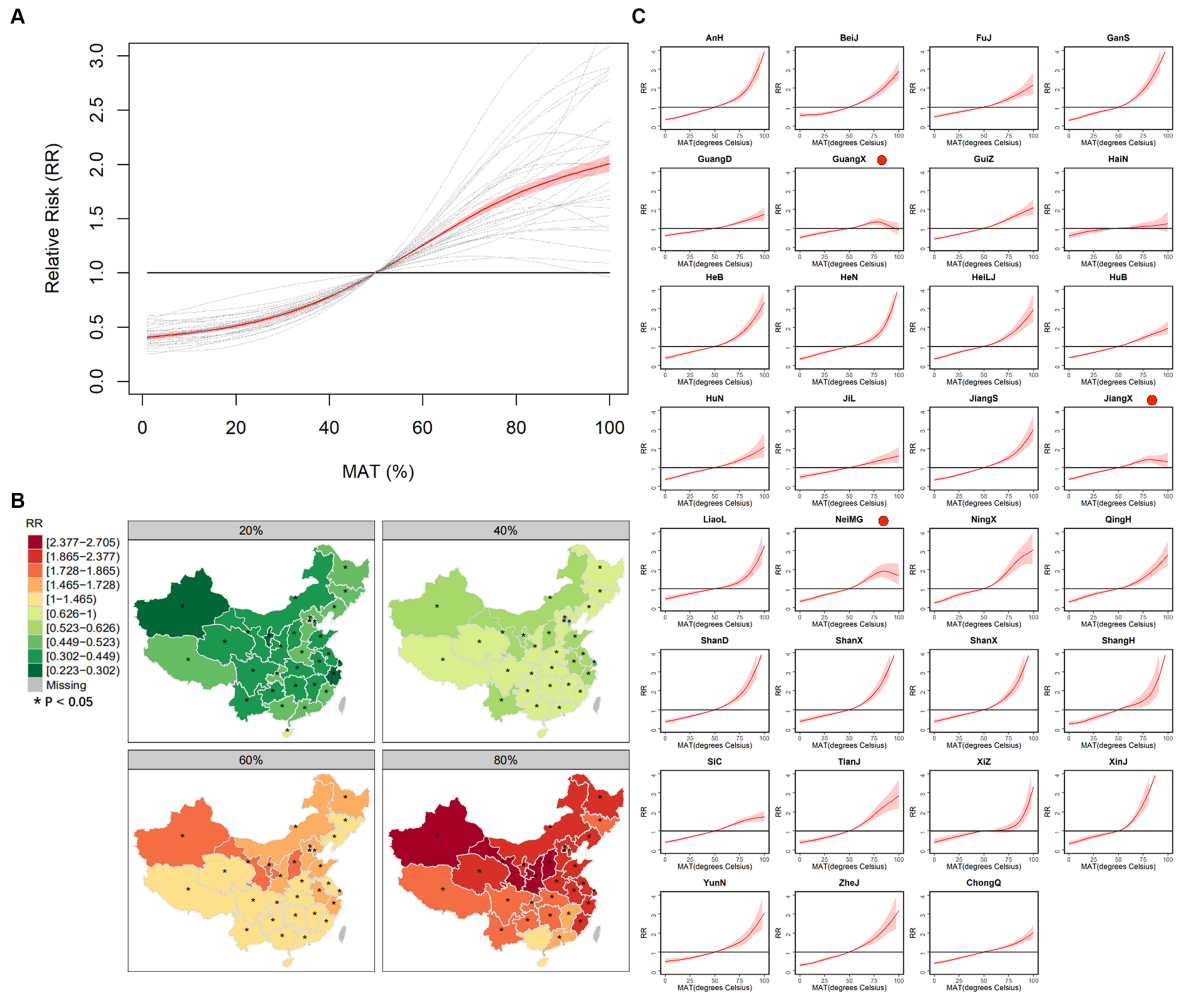
The curves of average association **(A)** and province-specific association between percentile MAT and BD **(C)** and spatial distribution of RRs of some representative percentile MAT, i.e., 20%, 40%, 60% and 80% **(B)**.

Figure 6 shows the association curves and spatial distribution of RR of relative MAT. As seen in Figure 6A, the association curves show a positive correlation, and most of their growth becomes flat for relatively high and low MATs, even though some of them show a negative correlation when the relative MAT reaches higher than 80%. Figure 6B shows the spatial distribution of relative risk: The risks of northern provinces are more sensitive to MAT, while Yunnan is always a high-risk area. The RRs of representative relative MAT and division of area of 31 provinces can be seen in Supplementary Table S3, and the maximum RR is 2.886 of 80% in Xinjiang, and the minimum RR is 0.333 of 20% in Shanghai. Figure 6C shows the province-specific association curves, and the curves of Guangxi, Inner Mongolia, and Jiangxi present an inverted “v” shape for the range of high relative MAT. In addition, most curves become steeper when the relative MAT reaches higher than 75%.

We calculated four indices, that is, the Deviance Information Criterion (DIC), the effective number of parameters (PD) in DIC, the Watanabe–Akaike information criterion (WAIC), and the logarithmic score (LS), and a smaller value meant a better model performance. As seen in Supplementary Table S1, LCAR outperformed meta-analysis. For absolute MAT, four indices of LCAR were lower than the meta-analysis approximately 4, 4, 43, and 5%, respectively. In particular, for WAIC, the LCAR had a significant improvement.

### 3.3 Attributable burden of BD due to high temperature

**Figure 7:**
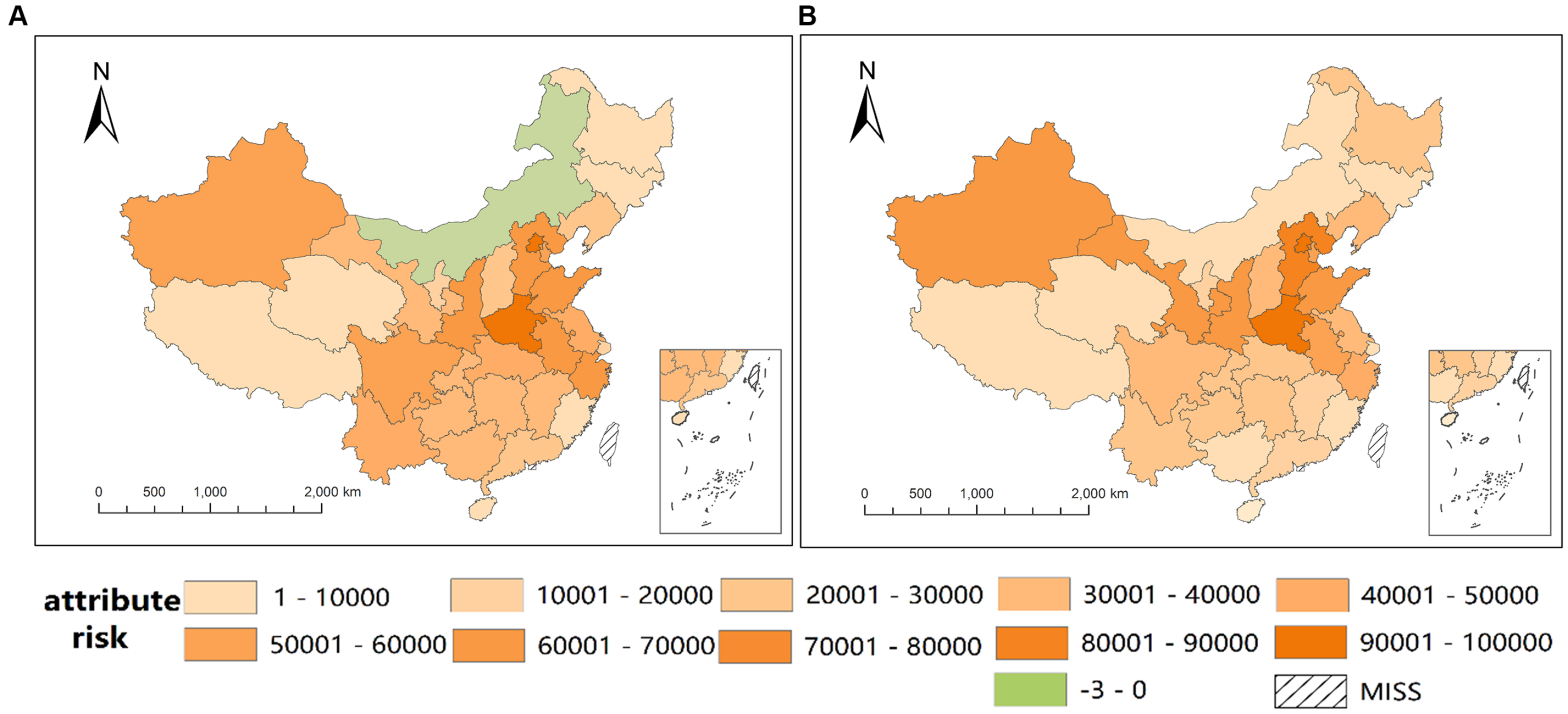
Spatial distribution of attribute number of BD cases due to high temperature of absolute MAT **(A)** and relative MAT **(B)**.

Based on the accurate and stable effect estimation of absolute and relative MAT by LCAR in the second stage, we calculate the attribute risk represented by a total number of BD cases due to high temperatures above the median temperature for 31 provinces. For the absolute MAT seen in Figure 7A, the maximum attributable case is approximately 97,681 in Henan, and the minimum is approximately 207 in Xizang, while the attributed risk in Inner Mongolia is negative. Comparing the eastern, central, and western parts of China, the attributable number of cases in each province is approximately 40,991, 42,025, and 26,947, respectively. For the relative MAT seen in Figure 7B, the maximum attribute risk is approximately 98,328 in Beijing, and the minimum is approximately 650 in Hainan.

## 4 Discussion

As the first study to consider the spatial autocorrelation of temperature-BD association by an LCAR-based model which has demonstrated the capacity to obtain a more accurate and stable estimation (9, 10, 16), we characterize the spatial distribution of spatially smoothed association and find that western provinces have a higher relative risk than eastern provinces. The specific curves indicate a positive correlation between temperature and BD, although some exhibit an inverted “V” shape under high temperatures. Subsequently, we delineate the more precise spatial distribution of the attributable number of cases.

In the first stage, in order to guarantee the sample size of each province, we collected monthly cases of BD with a minimum value of 73.41 in Hainan and adopted the GAM model to characterize the non-linear temperature-BD association. For the confounding factors, there are many other meteorological and social factors in addition to the rain, wind, humidity, and sun, and the associated forms are varied, depending on the objective of the study. According to the result of Moran’s *I*, there is a distinct spatial autocorrelation of association that the RRs of eastern provinces are mainly higher than those of western provinces and closed provinces have similar RRs, as seen in Supplementary Figure S1. Therefore, it is inappropriate for our study to employ meta-analysis regression, which ignores spatial autocorrelation.

In this study, we adopted two types of MAT, i.e., absolute MAT and relative MAT, which had different epidemiological values. For the abstract MAT, it was intuitive for epidemiology explanation that the same abstract MAT has the same association, which is consistent with the traditional epidemiological perspective and it was adopted generally in a relatively small area with a similar range of temperature. In particular, for some provinces with extreme climates, such as Tibet, absolute MAT is more appropriate to explain why they have higher or lower relative risk compared with other regions. For the relative MAT, the association is consistent in the same percentage of temperature of each province and it was generally used in a wide area, e.g., countrywide or nationwide, with a great variation of temperature. Therefore, we drew the average and specific association curves of the two types of MAT and exhibited the spatial distribution of RRs obtained by LCAR in the second stage.

For the absolute MAT, its average curve shows a generally increased relative risk with increased MAT, which is consistent with other studies (17, 18). In contrast, specific curves show different strengths of association, even an inverted “v” shape, which reveals the existence of heterogeneity in association across 31 provinces. Meanwhile, the spatial distribution of association indicates higher relative risks (RRs) in western provinces. This suggests a significant spatial correlation in these associations, which may be caused by different distributions of effect modifiers, particularly spatially correlated socioeconomic factors, which were proved in many studies. A study in Beijing, Tianjin, and Hebei indicated that socioeconomic factors, e.g., *per capita* GDP, population density, and rural population proportion, will affect the spatial distribution of BD (19) and might become effect modifiers of temperature. Similarly, a study conducted in urban and rural Hefei revealed that population density could modify the association between temperature and BD (20). Additionally, a study in Wuhan found that the central districts of cities are more likely to be high-risk areas for BD (21).

To intuitively exhibit the association for each province, we draw the specific curves with the same range of MAT and RR. As seen in Figure 4C, most curves show a positive correlation with a threshold of 10°C, and the curve becomes steeper when MAT reaches a higher value, which is consistent with other studies (22, 23), while the threshold is detected at 8°C in Denmark (24). According to previous studies, BD bacteria do not proliferate below 10°C, and their maximum growth rate is obtained at 37°C (25). Meanwhile, BD is mainly transmitted by the fecal-oral route through polluted food and water, and people are more likely to eat cold food such that the survival rate of *Shigella* on food will be increased at a refrigerated temperature (4°C) (26) and contact polluted water at a high temperature. As for the labeled curves of provinces with an inverted “V” shape, e.g., Guangxi and Jiangxi, extremely high temperatures will decrease the viability and spread of the bacteria, and inhibit bacterial growth in food and water. In contrast, people tend to take a holiday to Hainan, which has extreme heat in summer, increasing the transmission risk.

For relative MAT, the association curves show a positive correlation, with many plateauing at relatively high and low MAT levels. The phenomenon may arise because low MAT can restrict the growth and spread of the virus, while high MAT can limit outdoor activities, thus reducing the likelihood of infection within provinces. The spatial distribution of association highlights the greater sensitivity of northern provinces to MAT. Furthermore, it is more meaningful to examine the association within each province, rather than across provinces, particularly for those experiencing extreme temperatures, such as Hainan with extremely high temperatures and Xizang with extremely low temperatures. In contrast to the curves depicting absolute MAT, the curve for Hainan exhibits no significance above 50% MAT, while the curve for Xizang indicates a higher relative risk. The reason may be that the RRs of absolute MAT are calculated by a reference of 15°C, which is almost the minimum and maximum temperature of Hainan and Xizang, respectively, bringing about a misestimate of RR and its association.

Based on more accurate and stable estimations of association, we calculate the attribute number (AN) of BD cases due to high temperature to evaluate the attribute risk and characterize its spatial distribution. As seen in Figure 6A, the spatial distribution of AN of the absolute MAT shows that high-burden regions are mainly distributed in the middle and eastern areas, which is opposite to the spatial distribution of RRs. This is because the AN depends on the RR and population, and the population distribution of China follows a pattern where the east is densely populated and the west is sparsely populated (27). Meanwhile, the attributed risk of Inner Mongolia is negative, which means a protective effect of high temperature. Figure 6B, depicting AN based on relative MAT, showcases a similar spatial distribution, albeit with Inner Mongolia’s AN at 9507, contrasting with Figure 6A. In addition, the ANs of some other provinces in Figure 6B are also larger than those in Figure 6A, which may be due to the underestimation of RRs caused by an inappropriate reference of 15°C.

## 5 Perspectives

According to more accurate estimations of attribute risk and spatial distribution of association, medical institutions such as the Chinese Center for Disease Control and Prevention can reasonably distribute the resources of public health that the eastern and northern provinces and Xinjiang should be considered as risky regions and prevent and control BD promptly when the temperature increases in western provinces, whose risks are more sensitive to temperature. Meanwhile, according to the different thresholds in specific curves, policymakers can set different warning values for high temperatures. In addition, the local institutions are supposed to prepare sufficiently to prevent and control the coming heat because the curve of association between temperature and BD becomes steeper for a high temperature.

## 6 Limitations

Our study is the first to accurately characterize the spatial distribution of exposure-response association with a consideration of spatial correlation and evaluate the attributable BD cases. There are also some limitations in our study. First, due to the seasonal trend of BD, which mainly spreads in summer and autumn, the daily cases of some provinces in spring and winter have many missing values. Therefore, we analyzed monthly cases to analysis, and it was unable to characterize the lag correlation of BD on a small timescale, such as week or day. Second, the estimation of exposure-response association is only at the province level, and for the high-burden province, it is worthwhile for further studies to explore the spatial distribution of association in a high resolution, like city and county.

## 7 Conclusion

Through the utilization of the LCAR-based strategy, we achieved a more precise spatial distribution of the temperature-birth defect (BD) association and attributable cases across China. The spatial distribution of association exhibits notable heterogeneity, with the relative risks in western provinces demonstrating heightened sensitivity to MAT. The association curves generally show a positive correlation with a threshold at 10°C. Meanwhile, the spatial distribution of the attributed risk of MAT on BD reveals the high-risk areas in the eastern and northern provinces. Overall, these findings are conducive to guiding the reasonable distribution of resources for public health and the prevention and control of BD by medical institutions.

## Data availability statement

The original contributions presented in the study are included in the article/Supplementary material, further inquiries can be directed to the corresponding author.

## Ethics statement

Our study is a retrospective study involving previously collected data, such as information from the monitor system of infectious disease, and typically do not require ethics approval. Meanwhile, our study was constructed at the population level, which has no personal informations. Therefore, no confidential information was involved in this study and informed consent was not required.

## Author contributions

JW: Writing – review & editing, Conceptualization, Methodology, Writing – original draft. YW: Writing – review & editing, Investigation. WW: Writing – review & editing, Conceptualization, Funding acquisition, Methodology, Writing – original draft. YZ: Writing – review & editing, Data curation. JZ: Writing – review & editing, Data curation. YD: Writing – review & editing, Data curation. TZ: Funding acquisition, Writing – original draft, Writing – review & editing. FY: Writing – review & editing, Conceptualization, Funding acquisition, Writing – original draft. YM: Writing – review & editing, Writing – original draft. TS: Writing – review & editing, Data curation. KL: Methodology, Writing – review & editing.

## Footnotes

^1^http://en.chinacdc.cn/

^2^http://cdc.cma.gov.cn/
