# Supplementary material for: Using a Leroux-prior-based conditional autoregression-based strategy to map the short-term association between temperature and bacillary dysentery and its attributable burden in China
Source: Front Public Health. 2024 May 17;12:1297635. doi: 10.3389/fpubh.2024.1297635 (PMC11140140; doi:10.3389/fpubh.2024.1297635)
Supplement: Supplementary file 1 [file Data_Sheet_1.docx]

Supplementary Material

**Abbreviated names of study regions**

Anhui(AnH) Beijing(BeiJ) Fujian(FuJ) Gansu(GanS) Guangdong(GuangD) Guangxi(GuangX) Guizhou(GuiZ) Hainan(HaiN) Hebei(HeB) Henan(HeN) Heilongjiang(HeiLJ) Hubei(HuB) Hunan(HuN) Jilin(JiL) Jiangsu(JiangS) Jiangxi(JiangX) Liaoning(LiaoN) Inner Mongolia(NeiMG) Ningxia(NingX) Qinghai(QingH) Shandong(ShanD) Shanxi(ShanX) Shaanxi(ShanX) Shanghai(ShangH) Sichuan(SiC) Tianjin(TianJ) Xizang(XiZ) Xinjiang(XinJ) Yunnan(YunN) Zhejiang(ZheJ) Chongqing(ChongQ) Macau(MaC) Hong Kong(HongK) Taiwan(TaiW)


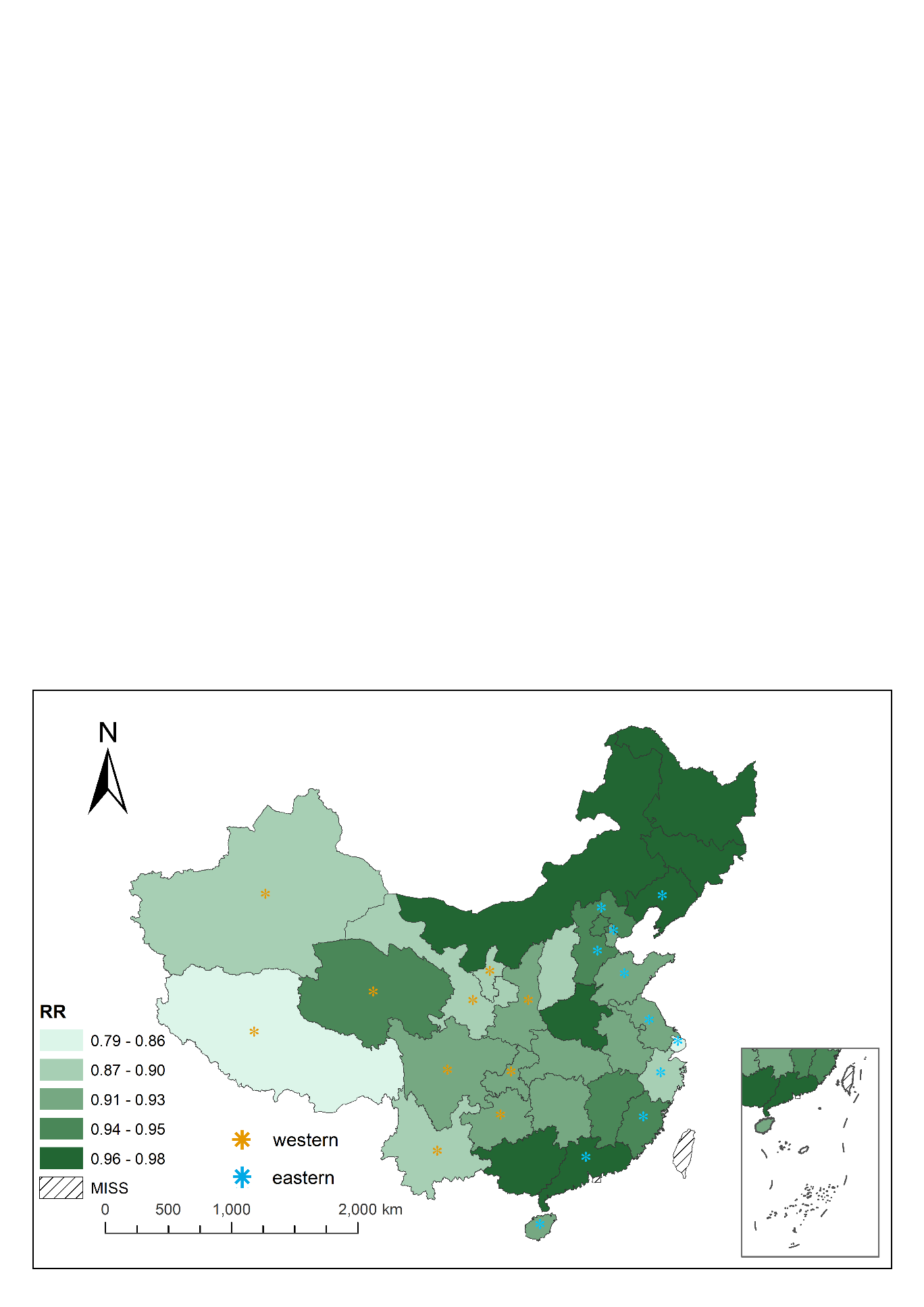


**Supplementary figure S1.The spatial distribution of RRs of 14°C in the first stage**

|  | Absolute MAT | | Percentile MAT | |
| --- | --- | --- | --- | --- |
|  | LCAR | META | LCAR | META |
| LS | 13.84 | 14.59 | 22.85 | 24.24 |
| PD | -36.10 | -34.77 | -70.99 | -68.41 |
| DIC | -38.77 | -37.36 | -73.75 | -71.19 |
| WAIC | -9.95 | -6.96 | -22.80 | -18.79 |

**Supplementary table S1.The mean of four indexes, i.e., LA, PD, DIC and WAIC calculated in LCAR and meta-analysis by two types of MATs.**

| Province | 5 | 10 | 20 | 25 | area |
| --- | --- | --- | --- | --- | --- |
| AnH | 0.489 | 0.673 | 1.506 | 2.297 | central |
| BeiJ | 0.562 | 0.720 | 1.405 | 1.826 | eastern |
| FuJ | NA | 0.756 | 1.409 | 2.090 | eastern |
| GanS | 0.436 | 0.610 | 1.550 | NA | western |
| GuangD | NA | NA | 1.238 | 1.726 | eastern |
| GuangX | NA | 0.767 | 1.353 | 1.802 | central |
| GuiZ | 0.517 | 0.672 | 1.543 | 1.960 | western |
| HaiN | NA | NA | 1.475 | 1.947 | eastern |
| HeB | 0.577 | 0.733 | 1.432 | 1.954 | eastern |
| HeN | 0.551 | 0.746 | 1.387 | 2.160 | central |
| HeiLJ | 0.626 | 0.791 | 1.224 | NA | central |
| HuB | 0.551 | 0.706 | 1.420 | 1.845 | central |
| HuN | 0.508 | 0.698 | 1.395 | 1.846 | central |
| JiL | 0.728 | 0.871 | 1.094 | NA | central |
| JiangS | 0.495 | 0.665 | 1.453 | 2.053 | eastern |
| JiangX | 0.517 | 0.717 | 1.370 | 1.729 | central |
| LiaoL | 0.688 | 0.826 | 1.302 | 1.949 | eastern |
| NeiMG | 0.555 | 0.753 | 1.025 | NA | central |
| NingX | 0.462 | 0.623 | 1.460 | NA | western |
| QingH | 0.494 | 0.690 | NA | NA | western |
| ShanD | 0.523 | 0.692 | 1.587 | 2.397 | eastern |
| ShanX | 0.523 | 0.660 | 1.644 | 2.312 | central |
| ShanX | 0.523 | 0.679 | 1.724 | 2.587 | western |
| ShangH | 0.435 | 0.560 | 1.760 | 2.360 | eastern |
| SiC | 0.488 | 0.639 | 1.484 | 1.749 | western |
| TianJ | 0.516 | 0.681 | 1.535 | 2.031 | eastern |
| XiZ | 0.515 | 0.594 | NA | NA | western |
| XinJ | 0.432 | 0.571 | 1.802 | 2.287 | western |
| YunN | NA | 0.686 | 1.871 | NA | western |
| ZheJ | 0.481 | 0.619 | 1.807 | 2.704 | eastern |
| ChongQ | 0.535 | 0.688 | 1.437 | 1.850 | western |

**Supplementary table S2.The relative risk of representative abstract MAT, i.e., 5, 10, 20 and 25℃, and division of area of 31 provinces.**

| Province | 20% | 40% | 60% | 80% | area |
| --- | --- | --- | --- | --- | --- |
| AnH | 0.453 | 0.748 | 1.296 | 1.978 | central |
| BeiJ | 0.550 | 0.762 | 1.316 | 1.903 | eastern |
| FuJ | 0.619 | 0.823 | 1.220 | 1.586 | eastern |
| GanS | 0.438 | 0.735 | 1.383 | 2.280 | western |
| GuangD | 0.675 | 0.858 | 1.153 | 1.373 | eastern |
| GuangX | 0.590 | 0.832 | 1.113 | 1.126 | central |
| GuiZ | 0.528 | 0.791 | 1.214 | 1.539 | western |
| HaiN | 0.784 | 0.943 | 1.063 | 1.141 | eastern |
| HeB | 0.532 | 0.786 | 1.300 | 1.954 | eastern |
| HeN | 0.549 | 0.812 | 1.271 | 2.047 | central |
| HeiLJ | 0.548 | 0.799 | 1.273 | 1.807 | central |
| HuB | 0.521 | 0.788 | 1.211 | 1.533 | central |
| HuN | 0.517 | 0.801 | 1.190 | 1.500 | central |
| JiL | 0.613 | 0.833 | 1.160 | 1.382 | central |
| JiangS | 0.448 | 0.742 | 1.264 | 1.779 | eastern |
| JiangX | 0.529 | 0.802 | 1.178 | 1.319 | central |
| LiaoL | 0.629 | 0.843 | 1.198 | 1.694 | eastern |
| NeiMG | 0.458 | 0.749 | 1.318 | 1.709 | central |
| NingX | 0.413 | 0.737 | 1.396 | 2.207 | western |
| QingH | 0.461 | 0.771 | 1.219 | 1.634 | western |
| ShanD | 0.497 | 0.759 | 1.364 | 2.260 | eastern |
| ShanX | 0.518 | 0.770 | 1.392 | 2.429 | central |
| ShanX | 0.530 | 0.785 | 1.355 | 2.358 | western |
| ShangH | 0.333 | 0.680 | 1.275 | 1.792 | eastern |
| SiC | 0.500 | 0.776 | 1.199 | 1.430 | western |
| TianJ | 0.451 | 0.728 | 1.355 | 1.947 | eastern |
| XiZ | 0.561 | 0.819 | 1.112 | 1.381 | western |
| XinJ | 0.393 | 0.697 | 1.530 | 2.886 | western |
| YunN | 0.490 | 0.761 | 1.217 | 1.538 | western |
| ZheJ | 0.399 | 0.708 | 1.338 | 1.932 | eastern |
| ChongQ | 0.545 | 0.807 | 1.161 | 1.433 | western |

**Supplementary table S3.The relative risk of representative relative MAT, i.e., 20%, 40%, 60% and 80%, and division of area of 31 provinces.**
